# BMC Clinical Pathology Reviewer Acknowledgment 2015

**DOI:** 10.1186/s12907-016-0024-x

**Published:** 2016-02-18

**Authors:** Elaine Zhang

**Affiliations:** BMC Clinical Pathology, BioMed Central, 233 Spring Street, New York, NY 10013 USA

## Abstract

The editors of BMC Clinical Pathology would like to thank all our reviewers who have contributed to the journal in Volume 15 (2015).

**Eshan Affan**

Australia

**Atif Ahmed**

USA

**Lara Alessandrini**

Italy

**Emyr Wyn Benbow**

UK

**Fiona Blows**

UK

**Camilla Böckelman**

Finland

**Annegien Broeks**

Netherlands

**Paul Cane**

UK

**Joshua Coleman**

USA

**Diego O. Croci**

Argentina

**Julie Cunningham**

USA

**Henrique De Amorim Almeida**

USA

**Ricardo Dos Reis**

Brazil

**Christina Ellervik**

Denmark

**Isabel Fonseca**

Portugal

**Andrea Galli**

Italy

**Jeff Garner**

UK

**Tomasz Gosiewski**

Poland

**Gaurav Gupta**

USA

**Sultan Habeebu**

USA

**Rui Henrique**

Portugal

**Alyson Hunter**

Ireland

**Jing Jiang**

China

**Ekaterina Jordanova**

Netherlands

**Kevin Kalinsky**

USA

**Janina Kulka**

Hungary

**Kari Kuulasmaa**

Finland

**Belinda Lategan**

Canada

**Chia-Hwa Lee**

Taiwan

**Hannah Leslie**

USA

**Kent Lewandrowski**

USA

**Xu-Yong Lin**

USA

**Deqin Ma**

USA

**Pam Michelow**

South Africa

**Sergio Morini**

Italy

**Dwight Oliver**

USA

**Giorgio Perino**

USA

**Ari Probandari**

Indonesia

**Lakshmy Ramakrishnan**

India

**U.S. Rao**

USA

**Madhur Ravikumara**

Australia

**Massimo Rugge**

Italy

**Isabel Scaletsky**

Brazil

**Robert Schmidt**

USA

**Vivekanand Singh**

USA

**Steven Smith**

USA

**Neil Spooner**

UK

**Jerzy Stanek**

USA

**Simona Stolnicu**

Romania

**Ryan Sullivan**

USA

**Karoly Szuhai**

Netherlands

**Alexandar Tzankov**

Switzerland

**Nicola Volpi**

Italy

**Semir Vranic**

Bosnia And Herzegovina

**Michael Whitehouse**

UK

**Dehua Yu**

China

